# Die materielle Kultur der Alchemie oder Wie sich wissenschaftsgeschichtliche Replikationen und buchwissenschaftliche Analysen ergänzen

**DOI:** 10.1007/s00048-022-00337-8

**Published:** 2022-07-12

**Authors:** Ute Frietsch

**Affiliations:** Josephinum – Ethik, Sammlungen und Geschichte der Medizin, Medizinische Universität Wien, Wien, Österreich

**William R. Newman 2019: *Newton the Alchemist: Science, Enigma, and the Quest for Nature’s “Secret Fire”*.** Princeton, NJ: Princeton University Press, geb., 537 S., € 37.20, ISBN: 978-0-691-17487‑7.

**Tara Nummedal 2019: *Anna Zieglerin and the Lion’s Blood: Alchemy and End Times in Reformation Germany*.** Philadelphia: University of Pennsylvania Press, geb., 304 S., 17 Abb., € 43.00, ISBN-13: 978-0-8122-5089‑3.

**Lawrence M. Principe 2020: *The Transmutations of Chymistry: Wilhelm Homberg and the Académie Royale des Sciences*.** Chicago, IL: The University of Chicago Press, geb., 504 S., 22 Abb., € 45.00, ISBN-13: 978-0-226-70078‑6; ISBN-10: 0‑226-70078‑x.

**Jennifer M. Rampling 2020: *The Experimental Fire: Inventing English Alchemy, 1300–1700*.** Chicago, IL: The University of Chicago Press, geb., 416 S., 19 Abb., € 31.50, ISBN-13: 978-0-226-71070‑9; ISBN-10: 0‑22671070‑x.

## Natürliches und Künstliches

Replikationen von historischen Rezepten und Experimenten sind unter anderem deswegen attraktiv, weil sie die moderne Trennung von Naturwissenschaft und Geisteswissenschaft relativieren: Wissenschaftsgeschichtlich ist es oftmals sinnvoll, mit Methoden beider „Kulturen“ zu arbeiten, zumal wenn es darum geht, vormoderne naturkundliche Produkte der Gelehrsamkeit zu analysieren. Es ist daher kein Zufall, dass sich gerade die Historiographie zur vormodernen Alchemie in den letzten Jahrzehnten als ein Gebiet der Wissenschaftsgeschichte etabliert hat, in dem philologische mit experimentellen Untersuchungen kombiniert werden. Im Zentrum steht dabei die materielle Kultur, wobei zunehmend auch die Buchkultur zum Gegenstand materialkundlicher Analysen wird. Manche Alchemiehistoriker*innen analysieren ihre Quellen mit buchwissenschaftlichen Methoden. Sie interpretieren sie nicht lediglich als Textzeugen, sondern untersuchen sie als dreidimensionale Artefakte: Provenienzen (die sich beispielsweise an Einbänden und Wasserzeichen zeigen), Formate (ein luxuriöser Folioband liest sich anders als ein Oktavbüchlein), Gebrauchsspuren (von Kratzern über Wasser- und Säurespritzer bis hin zu Brandlöchern), Annotationen (Anmerkungen, Diagramme und Skizzen von unterschiedlichen Händen), zwischen Autoren und Editoren ausgehandelte Methoden der Druckkunst (etwa die typographische Gestaltung idiosynkratischer Symbole) werden akribisch analysiert.

Mit einer doppelten, sowohl philologischen und buchwissenschaftlichen wie historisch-experimentellen Kompetenz überzeugen insbesondere Lawrence Principe und William Newman. Doch allen vier hier vorgestellten Publikationen gelingt es, mittels Analysen materieller Aspekte den einstigen Werkzeugcharakter ihrer Quellen nachvollziehbar zu machen. Jennifer Rampling widmet sich der Konstituierung einer nationalen „englischen“ Alchemie in der Zeit von 1300 bis 1700. Sie arbeitet mit Manuskripten, die oftmals unerschlossen sind, untersucht den Medienwechsel von der Handschrift zum Druck und repliziert zudem ein Experiment ihres Protagonisten George Ripley. Tara Nummedal analysiert Gerichtsakten aus den 1570er Jahren, die einen spektakulären Prozess gegen eine Alchemiker-Bande im Fürstentum Braunschweig-Wolfenbüttel repräsentieren. Sie reflektiert über die Gründe, die zu der lückenhaften Überlieferung der Akten geführt haben. Lawrence Principe widmet sich dem Chymiker Wilhelm Homberg und seiner Arbeit an der Académie Royale des Sciences und folgt seinem Protagonisten durch zehn Länder. Die letzte Textstufe von Hombergs unveröffentlichtem Manuskript *Élémens de chimie* spürt er im Boerhaave Archiv in St. Petersburg auf. William Newman wiederum wertet den Nachlass von Isaac Newtons alchemischen Manuskripten aus, der an der Indiana University digital ediert wird.^1^ Seine Publikation liefert Antworten auf die Frage, warum Newton ca. 30 Jahre lang zu Alchemie arbeitete und im Zuge dessen ca. eine Million Wörter notiert hat.

Positionierungen des 19. und frühen 20. Jahrhunderts haben sich so verzerrend auf die Geschichtsschreibung zur Alchemie ausgewirkt, dass die Arbeit in Archiven und Altbestandsbibliotheken heute als unerlässlich erscheint, wenn Alchemica unvoreingenommen analysiert werden sollen. Sie sind in der Regel ohnehin nicht modern ediert. Mittels der Replikation von alchemischen Experimenten wiederum lässt sich überprüfen, inwieweit die naturkundliche Arbeit manuell ausgeführt wurde und was sich daraus ergeben hat. Dass Chymia manuell praktiziert wurde, zeigt sich übrigens auch an ihren Imageproblemen, die denen der modernen Chemie ähneln, und sich unter Stichworten wie Fälschung, Schmutz und Gift zusammenfassen lassen.

Die vier Publikationen, die ich chronologisch, entsprechend der Reihenfolge ihrer Schwerpunkte bespreche, erschienen in rascher Folge bei den Verlagen Princeton University Press, University of Pennsylvania Press und University of Chicago Press. Dies dokumentiert, wie intensiv in den USA mit europäischen Alchemica gearbeitet wird. Die vier Autor*innen sind einschlägige Vertreter*innen eines leistungsstarken Netzwerks, das im englischsprachigen Raum besonders dicht geknüpft ist. Im Rahmen dieser Forschung wurden in den letzten zwei Jahrzehnten historiographische Standards erarbeitet, die sich unter anderem terminologisch geltend machen. So hat es sich mittlerweile durchgesetzt, die Geschichte der vormodernen Alchemie und der modernen Chemie als Kontinuum zu betrachten und dies durch Gebrauch von Begriffen wie „chymistry“ und „chymist“ anstatt „alchemy“ und „alchemist“ zu verdeutlichen.^2^ Entsprechende konzeptionelle Reflexionen werden im Deutschen durch die Verwendung von Quellenbegriffen wie „Chymia“ sowie durch Benennungen wie „Alchemiker“ zum Ausdruck gebracht. Aus der „chymistry“, so erläutert Principe pointiert, entstanden erst im 18. Jahrhundert Chemie einerseits und Alchemie andererseits (360–362). Für die These des Zusammenhangs von Alchemie und Chemie und die entsprechenden Untersuchungsansätze wurde in den letzten zwanzig Jahren die Bezeichnung „Neue Historiographie der Alchemie“ geprägt.^3^ Sie wird gelegentlich als einseitig – im Sinne einer Ausblendung der spirituellen, okkulten und hermetischen Gehalte der Alchemie – kritisiert.^4^

Dass es bei der „Neuen Historiographie der Alchemie“ jedoch keineswegs darum geht, historische Unterschiede einzuebnen, wird an Principes Untersuchungen zur *chrysopoeia* (Goldherstellung) deutlich. Principe bringt vielmehr eine Säule der Chemiegeschichte ins Wanken, indem er erklärt, dass die Ausgrenzung der *chrysopoeia* im 18. Jahrhundert weder aus wissenschaftlichen noch aus Gründen der Logik vorgenommen worden sei. Die *chrysopoeia* sei vielmehr der wissenschaftlich anspruchsvollste Part der historischen „chymistry“ gewesen, da Theorieproduktion und Experiment am engsten aneinandergekoppelt waren. Sie sei zwar ab den 1720er Jahren aus der Chymia ausgegrenzt worden und diese konnte sich daher in der Folge als Chemie konzipieren, während *chrysopoeia* als Alchemie diffamiert wurde. Gerade die bedeutendsten Gelehrten, wie Wilhelm Homberg und Isaac Newton, hätten ihre Forschung jedoch weiterhin, wenngleich nun gezwungenermaßen im Untergrund, geheim, privat beziehungsweise außerhalb der Öffentlichkeit, als *chrysopoeia* betrieben. Die Ausgrenzung der *chrysopoeia* sei in erster Linie von den staatlich etablierten Wissenschaftsverwaltungen betrieben worden, die um das Vertrauen in ihre Währungen fürchteten. Bedeutende Chymiker hingegen hätten auch im 18. Jahrhundert daran festgehalten, dass chymisches Gold so gut wie, wenn nicht besser als natürliches Gold sei. Principe verteidigt diese Gelehrten, indem er, mit Verweis auf eine Schrift Newmans, argumentiert:

While chrysopoeians insisted that chymically prepared gold was as good as (if not better than) natural gold, not everyone else acquiesced in this notion. (Such concerns have familiar counterparts in the modern world; for example, that an artificially prepared diamond is not a „real“ diamond, or that the „natural“ vitamin C in an orange is somehow different from the „artificial“ vitamin C in a tablet.) (236).

Forscher, die sich im 18. Jahrhundert der *chrysopoeia* widmeten, verstanden demnach, dass der Unterschied zwischen Natürlichem und Künstlichem letztlich nicht gegeben ist, während ihre Gegner wie moderne Essentialisten argumentierten. Diese These scheint mir nun durchaus in Widerspruch zu dem zu stehen, was man heute im Allgemeinen über Chemie und Chemiegeschichte zu wissen meint. Ich widme mich zunächst etwas ausführlicher dem buchwissenschaftlichen Zugang der Autor*innen und komme dann zurück auf Principes sowie Newmans These von der wissenschaftlichen Qualität der *chrysopoeia*.

## Eine englische Alchemie

Jennifer Rampling hat sich mit ihren Arbeiten zu dem mittelalterlichen englischen Alchemiker und Chorherren George Ripley und den ihm zugeschriebenen Schriftrollen einen Namen gemacht.^5^ Ripley steht auch im Zentrum ihrer wissenschaftsphilosophischen Publikation, welche die Erfindung einer „englischen“ Alchemie untersucht. Diese war Rampling zufolge maßgeblich durch die Reformation motiviert. Im Zuge der Auflösung der Klöster wurden mittelalterliche alchemische Quellentexte, die etwa an ihren Diagrammen leicht zu erkennen waren, als diabolisch oder papistisch gebrandmarkt und zum Teil zerstört. Ab der zweiten Hälfte des 16. Jahrhunderts erholte sich die Alchemie jedoch und gelangte zu einer Blüte. Die englischen Alchemiker besannen sich auf ihre eigene Tradition, anstatt sich weiterhin von den Theorien und Praktiken auf dem Kontinent leiten zu lassen. Sie reparierten, kompilierten, redigierten und edierten die alchemischen Bestände der aufgelösten Klöster und verfassten auf dieser Basis und mittels eigener Experimente neue Bücher. Eine spezifisch englische Tradition wurde konstruiert, indem man lateinische Texte ins Englische übersetzte, und sich vorrangig auf englischsprachige Alchemica bezog. Im Zuge dessen wurde Ripley zu einer zentralen Autoritätsfigur.

Rampling analysiert Ripleys Naturkunde und Naturphilosophie, die sich eine spezielle Auffassung des Mercurius (das „sericon“, 14), zum Ausgangspunkt nahm, und zeigt auf, dass diese Alchemie sowohl medizinisch (u. a. als Branntwein) wie transmutatorisch (u. a. als Quecksilber zur Gold- und Silberherstellung) genutzt wurde. Ripleys Alchemie habe sowohl aus Philologie wie aus Experimenten bestanden und sei als Lehre von drei Steinen der Weisen, einem pflanzlichen, einem mineralischen und einem animalischen, konzipiert worden. Sie sei philosophisch intelligibel gewesen und zudem als moralisch unanstößig und als praktisch effizient betrachtet worden. Für Ripleys Tradition war dabei typisch, dass das Vegetative betont wurde, das in der transmutativen Kraft des Quecksilbers wirksam sein sollte. Ripley vertrat die Theorie, dass alle Metalle aus Mercurius entstanden seien und sich folglich aus Quecksilber, unter Hinzunahme von Hilfssubstanzen, herstellen ließen. Er arbeitete mit Decknamen und der Technik der „dispersio“ (44), verstreute also wichtige Auskünfte gezielt über seine Schriften, um das Nacharbeiten seiner Prozesse zu erschweren.

Alchemie wurde in der Frühen Neuzeit nicht mehr länger in erster Linie von Mönchen, sondern beispielsweise von Prinzenerziehern und Bibliothekaren praktiziert. Eine Konstante war allerdings, dass sie sich oftmals Gönner suchen mussten. Die englische Alchemie wurde unter anderem von Elizabeth I., Edward III. und Henry VIII. protegiert, was die Fallhöhe scheiternder Alchemiker erklärt. Neben der Problematisierung der Goldfälschung, die bereits im 14. und 15. Jahrhundert akut war und bei der zwischen der *multiplicatio* des Materials und der falschen Prägung der Münzen unterschieden wurde (25–31), bildete sich ab dem 15. und 16. Jahrhundert durch die Rezeption neuer Autoren wie Pico della Mirandola und Agrippa von Nettesheim, und durch neue Theorieströmungen wie die christliche Kabbala eine magische Alchemie. Magie wurde unter anderem in Zusammenhang mit Hochverrat gebracht, und brachte die Adepten auf kürzestem Weg ins Gefängnis, während *chrysopoeia* sogar zur Entlassung beitragen konnte, da sich manche Regenten finanzielle Gewinne erhoffen. Die Ausübung der Alchemie stand dabei in engem Zusammenhang mit Phänomenen wie der Finanzkrise der 1540er Jahre. Rampling wertet mittelalterliche und frühneuzeitliche Textzeugen versiert in Hinblick auf Autorschaft, Authentizität, materielle Beschaffenheit und Tradierung aus. Besonders eingängig liest sich ihre Darstellung zu Edward Kelley und John Dee. Rampling arbeitet hier mit einem neuen Quellenfund, einem Manuskriptkonvolut der Universitätsbibliothek Leipzig, bei dem es sich um eine Sekretärsabschrift von Kelleys Gefängnisschriften handelt. Von allgemeinem Interesse ist auch ihre Darstellung zu Ashmoles *Theatrum Chymicum Britannicum*, das eine nationale Antwort auf Lazarus Zetzners *Theatrum Chymicum* darstellte. Rampling analysiert Annotationen in Ashmoles einst durchschossen gebundenem Handexemplar, das sich heute, in zwei Bände unterteilt, in der Bodleian Library befindet.

Rampling unternimmt eine beeindruckende Auswertung bislang relativ unbekannter und unzugänglicher Quellentexte. Auf ihre Darstellung von Ripleys Alchemie wird man sich in Zukunft stützen können. Das historische Konstrukt einer „englischen“ Alchemie leuchtet mir allerdings nur bedingt ein, zumal diese bereits von der Magie des 16. Jahrhunderts kompromittiert worden sein soll und um 1700 mit für England untypischen, magischen und antimonischen Interpretationen geendet haben soll. Rampling versteht Ripleys pseudo-lullische sericonische Alchemie als grundlegendes Muster der englischen Alchemie. Ashmoles magische Lektüre Ripleys war demzufolge für diese untypisch, wenngleich sie von Kelley und Dee inspiriert wurde. George Starkey wiederum habe den pflanzlichen Stein Ripleys in Anlehnung an den Paracelsisten Alexander von Suchten neu als Antimon konzipiert, womit er dessen Alchemie ebenfalls auf untypische Weise weiterentwickelte. Ob korpuskulartheoretische Modelle wie die Chemiatrie van Helmonts ebenfalls entscheidend waren für das Ende der englischen Alchemie, wird nicht deutlich. Insofern bleibt offen, ob Newton und Boyle, die mit Helmonts und Starkeys Ansätzen arbeiteten, zur englischen Alchemie zu rechnen sind. Robert Fludd, der unter anderem mit paracelsistischen Konzepten arbeitete, wird überraschenderweise überhaupt nicht genannt. So stellt sich die Frage, in welchem Verhältnis das Nationalbewusstsein der englischen Alchemie zu jenem der „deutschen“ Alchemie stand, die sich Paracelsus zur Gründungsfigur nahm. Diese Frage wird nicht expliziert, wenngleich die englische Alchemie unter anderem aufgrund des Entstehens dieser „deutschen“ Alchemie geendet zu haben scheint. Rampling benennt allerdings eine weitere interessante Parallele, nämlich die zur Erfindung einer frühen englischen Kirche, die dem römisch-katholischen Glauben vorausgegangen sei. Dieser nationale Plot sei ebenfalls in der Reformation geschaffen worden.

## Alchemie vor Gericht

Tara Nummedal nimmt sich in ihrer kulturhistorischen Monographie ein Korpus von Prozessakten aus den 1570er Jahren zum Gegenstand, das im Landesarchiv Wolfenbüttel erhalten ist. Die Akten dokumentieren einen spektakulären Fall von gescheiterter Patronage. Im Jahr 1575 ließ Herzog Julius von Braunschweig-Wolfenbüttel einige Alchemiker hinrichten, unter ihnen den einstigen Pfarrer Philipp Soemmering und die vermutlich durch Vergewaltigung und Kindsmord sozial gestrauchelte Anna Maria Ziegler, die nach eigener Aussage dem sächsischen Adel entstammte. Nummedal betrachtet Soemmering und Ziegler als die führenden Köpfe der Gruppe, da beide sich in Theorien und Praktiken der Alchemie einarbeiteten und dem Herzog entsprechende Angebote unterbreiteten. Ihre alchemischen Konzepte sind als Teil der umfangreichen Korrespondenzen und gerichtlichen Befragungen gelegentlich im Original oder in der Abschrift des Herzogs sowie in der Abschrift von Sekretären erhalten. Nummedal, die diesen Fall bereits in mehreren einschlägigen Publikationen behandelt hat,^6^ fokussiert insbesondere auf Theorien und Praktiken Zieglers. Sie bringt somit eine weibliche Stimme zum Sprechen, was insbesondere deswegen von Relevanz ist, weil Frauen – trotz ihrer alchemischen Tätigkeit im Kontext der Destillierkunst sowie der Haus- und Hofapotheken – in den gedruckten Alchemica des 16. Jahrhunderts nur schwach vertreten sind. Die Alchemie Zieglers konzentriert sich dabei vor allem auf die Manipulation der Fortpflanzung und berührt außerdem die *chrysopoeia*. Ziegler und Soemmering konzipierten ihre Alchemie in der paracelsistischen Tradition, sodass medizinisch-alchemische Aussagen mit magischen und christlich-kabbalistischen Konzepten einhergingen.

Die Gerichtsakten sind von der herzoglichen Familie offensichtlich selektiv überliefert worden, wie Nummedal zurecht betont. Nummedal arbeitet die Mythenbildung, die bereits zu Lebzeiten der Gruppe begann, in ihren Plot ein, der insofern dekonstruktive Züge hat. Es gelingt ihr, die Verzerrungen aufzuzeigen, die das Bild Zieglers prägten und hinter denen sich die historische Person lediglich anhand einiger Ego-Dokumente ausmachen lässt, von denen die meisten allerdings unter Folter generiert wurden. Während Ziegler sich selbst als Alchemikerin präsentierte und mittels eines Laboratoriums und eines Assistenten am Hof als solche tätig war, wurde sie im Verlauf des Gerichtsprozesses als Giftmischerin, Verführerin und Zauberin behandelt.

Die Stärke von Nummedals Darstellung besteht darin, dass sie ihre Quellen kritisch sondiert, sie jedoch auch sprechen lässt. Die Leser*innen werden so mit einem spektakulären Kriminalfall bekannt, der ungewöhnlich extensiv dokumentiert ist und der aufgrund der spezifischen Mischung von Themen wie Goldherstellung, Magie und Fortpflanzung von besonderem Interesse ist. Das Selbstbewusstsein Zieglers, die anhand des paracelsistischen Homunculus-Rezepts ein eigenes Rezept zur Herstellung eines neuen weiblichen Menschengeschlechts verfasste, widerspricht stereotypen Geschlechterkonzepten, wie sie in der Moderne auf vormoderne Gesellschaften projiziert wurden. Vergleicht man Nummedals Ansatz mit dem der weiteren hier besprochenen Monographien, ist allerdings auffällig, dass sie offenbar keine chemische Rekonstruktion der Rezepte in Erwägung gezogen hat. Somit ergibt sich der Eindruck, dass es sich bei den Rezepten Zieglers und Soemmerings um ausschließlich spekulative Texte handelt, was Nummedal jedoch nicht diskutiert.

## Eine Verteidigung der Chrysopoeia

Lawrence Principe wiederum schreibt in seiner Monographie zu Wilhelm Homberg die Geschichte der *chrysopoeia* in der Aufklärung neu. Principe macht deutlich, dass maßgebliche Forschung an der Académie Royale des Sciences um 1700 von Chymikern betrieben wurde, die sich der *chrysopoeia* widmeten. Sie waren sowohl an Experimenten wie an genuin chymischen Erklärungen interessiert, während Chymiker, die zu Pharmazie und Medizin arbeiteten, veraltete theoretische Erklärungen – wie die der drei paracelsischen Prinzipien Sulphur (Schwefel), Mercurius (Quecksilber) und Sal (Salz) – oftmals kritiklos als theoretischen Rahmen gelten ließen.

Principe schreibt die Biographie seines Protagonisten wie auch jene der Académie Royale des Sciences und der Chymia für die Zeit von ca. 1670 bis 1730. Er entheroisiert Homberg, dessen Vita seitens der Académie stilisiert worden ist. So überprüft er beispielsweise die Matrikeln und kommt zu dem Ergebnis, dass Homberg wohl lediglich einen juristischen, jedoch keinen medizinischen Abschluss an einer Universität absolvierte und somit in einer Zeit, in der dieser Begriff als Schimpfwort galt, als „Empiriker“ betrachtet werden konnte. Das theoretische Zentrum von Hombergs Forschung stellte dabei die Suche nach den chymischen Prinzipien dar. Homberg gelang es, Feuer und Säuren durch eine weniger destruktive Methode der Analyse zu ersetzen, indem er Gold und weitere Materialien mit einem Brennspiegel von Ehrenfried Walther von Tschirnhaus einschmolz und vaporisierte. Seine Interpretation dieser methodischen Möglichkeit bestand darin, Sonnenlicht als Ersatz für Feuer und Säure zu begreifen und es als Sulphur beziehungsweise sulphurhaltig zu betrachten.

Hombergs Assistent Étienne-François Geoffrey arbeitete sich in Johann Joachim Bechers und Georg Ernst Stahls Konzepte ein, die auch Homberg in seine Chymia zu integrieren versuchte. Während Stahl die drei Prinzipien durch Phlogiston ersetzte, nahm bei Homberg der Sulphur die zentrale Position ein. Homberg sowie Geoffrey vertraten die Auffassung, dass auch bei Prozessen der Calcination, etwas – nämlich Sulphur – hinzukam. Homberg war davon auch deswegen überzeugt, weil er methodisch mit der Waage arbeitete und beim Prozess des Verbrennens Gewichtszunahmen feststellte. In seinen *Élémens de chymie* versuchte er, ein neues transmutatorisches System der Chymia zu entwerfen, dessen Rahmen seine Sulphur-Theorie des Lichts bildete.

Principe schildert den problematischen Status der Chymia im Zusammenhang mit der „affaire des poisons“ (Giftaffäre) der späten 1670er Jahre sowie anhand von Akten der Bastille. Er arbeitet eine Affäre um den gebürtigen Neapolitaner Etienne Vinache heraus, die Verflechtungen zwischen Hombergs Laboratorium und kriminalisierten Pariser Chymikern verdeutlicht und dokumentiert, dass Chymia von der Pariser Polizei um 1700 als Vorwand für Giftmischerei und Geldfälschung betrachtet wurde. Selbst die Finanzminister hofften jedoch, gegebenenfalls echte Kompetenzen der *chrysopoeia* in Dienst nehmen zu können. Die Fähigkeiten der Chymiker zur Laboratoriumsarbeit wurden daher in der Haft examiniert. Principe betont, dass *chrysopoeia* zwar in den 1720er Jahren von Seiten der Académie in offiziellen Verlautbarungen abgelehnt wurde, dass hierfür jedoch die politische Lage ausschlaggebend war. Chymiker der Académie betrieben ihre Versuche auf dem Gebiet der *chrysopoeia* daraufhin quasi privat beziehungsweise im Untergrund, ohne sie weiterhin zu publizieren. Nach Hombergs Tod arbeiteten Newton und Lavoisier weiter an Hombergs Experimenten mit dem Brennglas. Bei Newton gingen die Ergebnisse in die chemischen Teile seiner *Opticks* (1717) ein. Bei Lavoisier waren sie Teil der Experimente, die zur Entdeckung des Sauerstoffs führten.

Principe widmet sich sowohl den Experimenten der Chymiker wie ihren Handschriften und gedruckten Publikationen. Er spürt kaum bekannte sowie verlorengeglaubte Manuskripte auf und vergleicht unterschiedliche Stufen und Versionen der Texte. Sein Buch liest sich sehr unterhaltsam, weil er die abenteuerlichen Aspekte sowohl der Quellen wie der eigenen Forschung thematisiert. Principe gelingt es zudem, die einzelnen Experimentalanordnungen auf völlig nachvollziehbare Weise zu verbalisieren und zu explizieren und sie so als Argumente zu präsentieren. Mit seiner Verteidigung der *chrysopoeia* plädiert er implizit dafür, nicht nur Chymia, sondern Chemie insgesamt als form- und veränderbare Form der Naturforschung zu begreifen, deren praktischen Arbeiten auch heute philosophisch anspruchsvolle Fragestellungen zugrunde liegen können.^7^

## Was suchte Newton in der Alchemie?

Die Relevanz von William Newmans Buch zu Newtons Alchemie ist angesichts des Renommees des historischen Protagonisten unmittelbar ersichtlich. Wie seit der Auktion von Newtons Manuskripten bei Sotheby im Jahr 1936 allgemein bekannt ist, widmete sich der „Vater“ der klassischen Physik mehr als dreißig Jahre lang der Alchemie. Er teilte seine alchemischen Manuskripte allerdings nur mit sehr engen Vertrauten und publizierte sie nicht. Newmans Publikation basiert auf den Ergebnissen der digitalen Edition dieser Manuskripte an der Indiana University, sowie auf seinen Replikationen von Newtons alchemischen Experimenten, die er in Farbfotografien dokumentiert. Die Gestalt seines Buches erinnert dabei an das Folioformat frühneuzeitlicher Werke – nicht zuletzt, weil ein sehr breiter Rand, wohl für Marginalien der Leser*innen, freigelassen ist.

Newman wendet sich in seiner Darstellung zunächst gegen die These, dass Newton Alchemie aus religiösen Bedürfnissen betrieben habe. Newman zufolge sind Newtons Methoden in seinen kirchengeschichtlichen und in seinen alchemischen Schriften völlig unterschiedlich. Wenn beide Manuskriptarten auch gemeinsam veräußert und daher in den letzten Jahrzehnten aufeinander bezogen wurden, haben sie doch keinen gemeinsamen konzeptionellen Rahmen. Newman weist zudem die These zurück, dass Newton durch seine alchemischen Studien auf sein Konzept der Gravitation gebracht worden sei. Von Bedeutung seien sie vielmehr für seine Optik gewesen.

Newton begann in den 1660er Jahren, zunächst als Student, mit seiner alchemischen Lektüre. Er stieß rasch auf Boyles, Sendivogius’ sowie Starkeys Untersuchungen zur *chrysopoeia*. 1669 kaufte er sich Laboratoriums-Equipment sowie die sechsbändige Ausgabe von Zetzners *Theatrum chemicum*. Damit war der Grundstein für seine alchemische Arbeit in seinem privaten Labor im Trinity College gelegt. Gegen Aristoteles’ Konzept der perfekten homogenen Mischung (mixis) sowie gegen die scholastische Position, dass die Bestandteile der Materie in dieser kosmologischen Mischung unwiederbringlich zerstört würden, argumentierte Boyle unter anderem in Anlehnung an van Helmont, dass Materie aus Korpuskeln bestehe, die in ihr unverändert erhalten blieben. Chymiker konnten daher mit Säuren und Metallen eine perfekte Mischung herstellen und die Prozesse daraufhin wieder umkehren. Newton griff dieses alchemische Konzept der Korpuskulartheorie auf und wandte es auf das Licht an. Gegen eine 2000-jährige Tradition, sowie in Auseinandersetzung mit den Konzepten Hombergs zum sulphurhaltigen Sonnenlicht, stellte er fest, dass das weiße Sonnenlicht heterogen (eine Mischung) war und aus Spektralfarben gebildet wurde. Newton vertrat die Auffassung, dass das Sonnenlicht vor der Fraktion in Spektralfarben und nach der Refraktion zu weißem Licht immer dasselbe sei. Newton sah sich hierin, Newman zufolge, von Boyle bestätigt, der die Auffassung mancher mittelalterlicher Alchemiker teilte, dass zwischen künstlichem und natürlichem Gold kein Unterschied bestehe.

In seiner Auswertung älterer alchemischer Schriften nutzte Newton insbesondere die Technik des Florilegiums. Wo Newtons Lektüre der Tradition endete und seine eigene Alchemie begann, lässt sich daher selbst mit digitalen Methoden nur schwer eruieren, wenngleich er – ähnlich heutigen Editoren – gelegentlich eckige Klammern für seine Interpretationen verwendete. Newton setzte seine eigenen alchemischen Ergebnisse allerdings einer rigorosen experimentellen Überprüfung aus. Da sie dieser nicht standhielten, gestand er sich ein, dass er zwar wohl ein auserwählter Adept war, die entsprechenden Resultate jedoch noch nicht erzielt hatte. Newton ging es dabei, Newman zufolge, insbesondere um eine Untersuchung des Aufbaus der Materie und um eine Naturphilosophie, die im Unterschied zu Descartes’ Philosophie die vegetativen Prozesse umfasste. Bei Newton bekamen die alchemischen Reflexionen zudem eine hylozoistische Ausrichtung. So betrachtete er die Erde als ein Lebewesen, das Äther ein- und ausatme (64–82, 136–149). Ab Mitte der 1670er Jahre ersetzte er seine von Sendivogius inspirierte Forschung zum Luft-Salz durch eine Sulphur-Theorie der Luft beziehungsweise des Lichts, womit er sich gegen die Phlogiston-Theorie entschied. Newtons private Forschungen zur *chrysopoeia* überschnitten sich mit seinen physikalischen Forschungen nicht nur auf dem Gebiet der Optik, sondern auch in Hinblick auf seine Aussagen zum Äther, zur Verbrennung, zur Mikrostruktur der Materie, zu Fermentation und Putrefaktion sowie zu den Affinitäten (436). Die Unterscheidung von öffentlicher physikalischer Forschung und privater chymischer Forschung entsprach dabei, Newman zufolge, nicht der Unterscheidung von praktisch versus spirituell. Privat sei vielmehr das geblieben, was als riskant betrachtet wurde, während Newton öffentlich machte, was er als sicher betrachten konnte.

Newmans Publikation ist für die Newton-Forschung absolut einschlägig. Sie stößt allerdings auf sprachliche Grenzen. Die Anfangs- und Endkapitel, die unter anderem frühere Forschungsansätze, Newtons Weg zur Alchemie sowie dessen Kooperationen und Ergebnisse schildern, lesen sich brillant. Der lange mittlere Teil rekonstruiert Newtons alchemische Forschungen allerdings äußerst akribisch anhand eines *close readings* und stellt sich als ein Archiv von Belegen dar. Die akribische Dokumentation von Newtons alchemischen Kompilationen sowie Versuchsanordnungen ist zwar für die Newton-Forschung überaus verdienstreich und unverzichtbar, lässt sich jedoch – ähnlich wie heutige chemische Formeln – nicht umstandslos lesen, zumal Newtons einsame Arbeiten oftmals in Sackgassen endeten. – In einem Online-Talk zu seinem Buch hat Newman ergänzt, dass Newtons Alchemie als eine Art „nuclear physics“ verstanden werden kann, bei der es darum gegangen sei, im chymischen Labor Materie-Partikel kleiner, sublimer und volatiler und somit aktiver zu machen.^8^

## Buch und Experiment

Die moderne Entgegensetzung von Geisteswissenschaften und Naturwissenschaften hat das historische Buchwissen und das empirisch naturkundliche Wissen in einen Gegensatz gebracht, der als fundamental erscheint. Auf historischen Darstellungen zur Chymia sind jedoch oftmals Bücher zu sehen, die im Laboratorium verfasst oder zu Rate gezogen werden – und das Labortagebuch ist auch als Praktik des 20. und 21. Jahrhunderts bekannt. Die eingangs benannte Problematik der fundamentalen Unterscheidung zweier Wissenschaftskulturen bildet den modernen Ausgangspunkt, von dem sich die aktuelle Forschung zur Alchemiegeschichte sowohl konzeptionell wie auch methodisch löst, indem Buch- und Experimentalkultur aufeinander bezogen und beide als materielle Kulturen ausgewertet werden. Im Ergebnis erscheint Chymia als hoch komplex, weil sie – unter anderem da sie spirituell, okkult und hermetisch verfasst war – sowohl naturkundliche wie philologische Kompetenzen erforderte. Alchemiegeschichte stellt sich als eine Chemie für Bibliophile dar, in der sich die materiellen Kulturen der Schrift und des Buches und die naturkundlichen Aussagen sowie Experimente zum Aufbau der Materie gleichermaßen sondieren lassen. Auf diese Weise wird deutlich, dass Chymia zurecht als Teil der (Natur‑)Philosophie betrachtet wurde – und dass Naturwissenschaften der Gegenwart (etwa Chemie, Physik, Pharmazie und Medizin) in ihrer disziplinären Verselbständigung als historische Antworten auf manche ihrer Fragen interpretiert werden können. Die Interpretation der (Al‑)Chemiegeschichte als Kontinuum trägt dazu bei, dass sie sich besser in beide Richtungen lesen lässt.

## Footnotes

1 William R. Newman (Hg.): http://www.chymistry.org (2005–2022).

2 Newman & Principe (1998).

3 Zum Konzept der „New Historiography of Alchemy“ vgl. Principe (2004).

4 Vgl. Vickers (2008).

5 Vgl. u. a. Rampling (2014).

6 Vgl. u. a. Nummedal (2007).

7 Ein ähnliches Anliegen vertreten Isabelle Stengers und Bernadette Bensaude-Vincent, die es allerdings mit einer Kritik am monotonen Berufsalltag moderner Chemiker*innen verbinden (1993).

8 Philosophical Cafe – Cafeneaua filosofica (Hg.): https://www.youtube.com/watch?v=HxmT4oEBoLE (03.11.^2020^), Länge 16:59, hier 7:44–9:40 (aufgerufen am 20.05.2022).
